# A very high prevalence of hepatitis C virus infection among patients undergoing hemodialysis in Kosovo: a nationwide study

**DOI:** 10.1186/s12882-018-1100-5

**Published:** 2018-11-03

**Authors:** Xhevat Jakupi, Jana Mlakar, Maja M. Lunar, Katja Seme, Ibrahim Rudhani, Lul Raka, Adriana Vince, Mario Poljak

**Affiliations:** 1 Department of Microbiology, Kosovo National Institute of Public Health, Rrethi i Spitalit, p.n, 10000 Prishtina, Kosovo; 2 Institute of Microbiology and Immunology, Faculty of Medicine, University of Ljubljana, Zaloška 4, 1000 Ljubljana, Slovenia; 3 Clinic for Nephrology, University Medical Center of Kosovo, Bulevardi i Dëshmorëve, p.n, 10000 Prishtina, Kosovo; 4 Faculty of Medicine, Hasan Prishtina University of Prishtina, Bulevardi i Dëshmorëve, p.n, 10000 Prishtina, Kosovo; 5 Dr Fran Mihaljević University Hospital for Infectious Diseases, Mirogojska 8, 10000 Zagreb, Croatia

**Keywords:** Hepatitis C virus, Hemodialysis, Infection, Infection control, Kosovo

## Abstract

**Background:**

Patients on hemodialysis are at high risk for hepatitis C virus (HCV) infection if measures for effective control of HCV infection in the hemodialysis environment are not implemented. Whereas in developed countries isolated small-scale outbreaks of HCV in hemodialysis units are occasionally reported, HCV transmission in the hemodialysis environment still represents a substantial problem in low-resource countries. This study systematically assessed the prevalence of HCV infection among all patients at all hemodialysis centers in Kosovo, determined the HCV genotype distribution, and reviewed the main risk factors associated with HCV infection in this group of patients.

**Methods:**

From January to March 2013, blood samples from all patients undergoing hemodialysis at all seven hemodialysis centers in Kosovo were collected. The samples were screened for the presence of anti-HCV antibodies, and seropositive samples were also tested for HCV RNA. Genotyping was performed by sequencing the core region of the HCV genome. Subsequently, face-to-face interviews were conducted with consented patients attending hemodialysis in December 2015 and with the management of all hemodialysis centers in Kosovo.

**Results:**

The overall seroprevalence of HCV infection among hemodialysis patients in Kosovo was 53.0% (354/668), ranging from 22.3 to 91.1% at different centers. HCV RNA was detected in 323/354 (91.2%) seropositive patients. The most frequent HCV genotype was genotype 1a (62.2%), followed by genotypes 4d (33.1%), 1b (4.0%), and 2c (0.7%). The duration of hemodialysis and receiving dialysis at more than one center were identified as independent significant predictors of anti-HCV positivity. Shortage of staff, lack of resources, and inconsistent use of hygienic precautions and/or isolation strategies were observed.

**Conclusions:**

The prevalence of HCV infection among hemodialysis patients in Kosovo is extremely high. The relatively low prevalence of HCV infection in the general population, predominance of two otherwise rare HCV genotypes among hemodialysis patients, and longer history of hemodialysis as a predictor of HCV infection all indicate nosocomial transmission due to inappropriate infection control practices as the main transmission route.

**Electronic supplementary material:**

The online version of this article (10.1186/s12882-018-1100-5) contains supplementary material, which is available to authorized users.

## Background

Hepatitis C virus (HCV) is one of the leading causes of acute and chronic hepatitis, cirrhosis of the liver, hepatocellular carcinoma, and related death [1, 2]. Up to 1% of the world’s population is estimated to be infected with HCV, corresponding to more than 71 million people with a viremic infection [3]. The prevalence of HCV-specific antibodies in the general population varies from less than 1.5% in North America and some parts of South America to over 3.5% in some regions of Africa and Asia [3]. In developed countries, most of the incident hepatitis C cases are due to acquisition and transmission among people that inject drugs, whereas in developing countries healthcare-associated transmission due to a poor standard of infection control and prevention remains the main route for HCV transmission [4, 5]. The largest documented iatrogenic HCV transmission occurred in Egypt due to mass population-based antischistosomal treatment involving administration of tartar emetic injections from 1960s to 1980s [6]. Patients on hemodialysis are at higher risk for HCV infection if measures for effective control of HCV infection in the hemodialysis environment are not implemented. Whereas in developed countries isolated small-scale HCV outbreaks in hemodialysis units are reported only occasionally, HCV transmission in the hemodialysis environment still represents a substantial problem in low-resource countries [7–9].

Kosovo is a small lower-middle-income country with 1.7 million inhabitants in southeastern Europe that has recently gone through war and postwar difficulties. Data on prevalence of HCV infection in Kosovo, and particularly among hemodialysis patients, are scarce. The seroprevalence of HCV infection was first assessed among Kosovar blood donors between 2000 and 2003 and was found to be 0.3% (206/70,348) [10]. The anti-HCV prevalence in the general population was studied in a single region in Kosovo in 2005 and reported to be 0.5% (7/1287), whereas the seroprevalence in hemodialysis patients from the same region in the same period was 86.4% (57/66) [11, 12]. In 2008 all patients treated at hemodialysis centers were tested and 42.9% (250/583) were found to be anti-HCV positive [13].

This study systematically assessed the prevalence of HCV infection among all patients at all hemodialysis centers in Kosovo, determined the HCV genotype distribution, and reviewed factors associated with HCV transmission for the first time in order to obtain baseline data before implementing the infection control measures and treatment strategies necessary to prevent the further spread of HCV infection in this specific healthcare setting.

## Materials and methods

All 668 patients that were treated with hemodialysis between January and March 2013 at all seven hemodialysis centers in Kosovo were included in this study. Blood samples were obtained by venipuncture at dialysis centers and were first screened for the presence of antibodies against HCV, using the ELISA anti-HCV test (Axiom, Bürstadt, Germany), following the manufacturer’s instructions. All samples that tested anti-HCV positive were also tested for HCV RNA using the COBAS AmpliPrep/COBAS TaqMan HCV Qualitative Test, v2.0 (Roche Molecular Diagnostics, Meylan, France).

HCV genotyping was performed in all viremic samples by sequencing the core region of the HCV genome, as previously described [14]. Briefly, viral nucleic acid was extracted from 200 μl of patient’s plasma samples, using the MagnaPure Compact Nucleic Acid Isolation kit I on the MagNA Pure Compact instrument (Roche Diagnostics, Mannheim, Germany) and amplified using SuperScript^TM^ III One-Step RT-PCR System with Platinum® Taq High Fidelity (Invitrogen, Carlsbad, CA, USA) and primers Sc2/Ac2 [14]. If no polymerase chain reaction (PCR) product was obtained, nested PCR with S7/A5 primers and the FastStart High Fidelity PCR System (Roche Diagnostics) was employed [14]. The acquired 441 bp or 355 bp amplicons of the HCV core region were purified with the addition of enzymes Exonuclease I and FastAP (Thermo Fisher Scientific, Waltham, MA, USA) and sequenced using a BigDye Terminator v3.1 Cycle Sequencing Kit (Applied Biosystems, Foster City, CA, USA) with the nested S7 primer. A BigDyeXTerminator Purification Kit (Applied Biosystems) was used for the removal of unincorporated dye terminators from the sequencing reactions. Sequencing was performed on the 3500 Genetic Analyzer (Applied Biosystems), and the HCV genotype determined using the NCBI genotyping tool [15], with the addition of genotype reference set 2012, retrieved from HCV Sequence Alignments [16].

In addition, all patients receiving hemodialysis at seven centers in Kosovo in December 2015 were invited to participate in the study and face-to-face interviews were conducted with those patients who signed written informed consent. The following information was collected: age, sex, education, employment status, duration of hemodialysis, location of hemodialysis, blood transfusion, surgical and dental interventions, and other risk factors (see Additional file 1: Table S1). Anti-HCV test results for the patients of the 2015 cohort were obtained from their medical records. Interviews were conducted with the management of hemodialysis centers with an emphasis on infection control and issues related to carrying out hemodialysis (availability of resources and staffing) (see Additional file 2: Table S2).

The statistical analysis was conducted using SPSS software, version 17.0. Data were expressed as mean ± standard deviation (*SD*), median and inter-quartile range, or frequencies, as appropriate. Differences between subgroups were assessed using a χ^2^ (chi-squared) test, Fisher’s exact test, Student’s *t*-test, and an analysis of variance test. The backward Poisson regression was used to assess the independent effect of a variable, and the prevalence ratios and their respective robust 95% confidence intervals were calculated. The Wald test was used as a statistical test. A *p*-value of less than 0.05 was considered statistically significant.

## Results

As shown in Table 1, the overall seroprevalence of HCV infection among 668 hemodialysis patients in Kosovo was 53.0% (354/668). The highest seroprevalence was observed at Center 7 (91.1%) (Table 1).

**Table 1:** HCV seropositivity, HCV RNA positivity, and distribution of HCV subtypes in hemodialysis patients in Kosovo in 2013

Hemodialysis center	Patients tested for aHCV *n*	HCV seropositivity *n* (%)	HCV RNA positivity *n* (%)	Subtype 1a *n* (%)	Subtype 1b *n* (%)	Subtype 2c *n* (%)	Subtype 4d *n* (%)	Total genotyped *n* (%)
Center 1	188	42 (22.3)	39 (92.9)	15 (46.9)	3 (9.4)	0	14 (43.8)	32 (82.1)
Center 2	68	46 (67.6)	42 (91.3)	25 (75.8)	1 (3.0)	0	7 (21.2)	33 (78.6)
Center 3	75	51 (68.0)	45 (88.2)	10 (24.4)	1 (2.4)	0	30 (73.2)	41 (91.1)
Center 4	163	112 (68.7)	102 (91.1)	55 (63.2)	1 (1.1)	1 (1.1)	30 (34.5)	87 (85.3)
Center 5	39	13 (33.3)	9 (69.2)	5 (100)	0	0	0	5 (55.6)
Center 6	79	39 (49.4)	38 (97.4)	26 (78.8)	4 (12.1)	1 (3.0)	2 (6.1)	33 (86.8)
Center 7	56	51 (91.1)	48 (94.1)	35 (79.5)	1 (2.3)	0	8 (18.2)	44 (91.7)
Total	668	354 (53.0)	323 (91.2)	171 (62.2)	11 (4.0)	2 (0.7)	91 (33.1)	275 (85.1)

HCV RNA was detected in 323/354 (91.2%) seropositive hemodialysis patients. Out of 323 viremic patients, 275 (85.1%) were successfully genotyped.

Nationwide, the most frequent HCV genotype among hemodialysis patients was genotype 1a (62.2%) followed by genotypes 4d (33.1%), 1b (4.0%), and 2c (0.7%) (Table 1). No other HCV genotypes were identified.

The distribution of HCV genotypes differed significantly among centers (Table 1). Subtype 1a was the most prevalent at all but one center, ranging from 24.4% at Center 3 to 100% at Center 5. The HCV genotype 4d was the most prevalent (73.2%) at Center 3 and was the second most prevalent HCV genotype at the remaining centers except Center 6, where it was the third most prevalent after genotypes 1a and 1b. HCV genotype 1b was detected in a total of only 11 patients. It was found at all centers except Center 5. HCV genotype 2c was found in two hemodialysis patients only, one each at Center 4 and Center 6 (Table 1).

Out of 708 patients receiving hemodialysis in Kosovo in December 2015, 618 (87.3%) signed written informed consent and were included in the survey. Of these 618 patients, 244 (39.5%) were anti-HCV positive. This cohort of patients differed from the initial HCV prevalence study; namely, 477 (77.2%) of the patients also participated in 2013.

In the study population, 51.6% were males, with a mean age of 56.3 (± 12.7 years; Table 2). The majority of patients were married (83.2%), 54.5% were unemployed, and 34% were retired. The level of education of the patients was as follows: none (15.5%), primary education (39.6%), secondary education (32.3%), and higher education (9.7%). Nine (1.5%) dialysis patients were healthcare workers (data not shown).

**Table 2:** Association of variables with anti-HCV positivity among patients on hemodialysis in December 2015 in Kosovo, as determined by univariate and multivariate logistic regression analysis

Explanatory variable	*n*	%	Anti-HCV positive (%)	Unadjusted PR	CI 95%	*p*-value	Adjusted PR	CI 95%	*p*-value
Sex									
Female	299	48.4	42.8	ref (1)		0.498	ref (1)		0.480
Male	319	51.6	36.4	0.955	0.836–1.910		0.978	0.818–1.170	
Age (years) Mean ± *SD* 56.3 ± 12.7				0.985	0.972–0.998	0.021*	0.997	0.992–1.003	0.327
Age (years)									
≤ 39	52	8.4	51.9						
40–49	96	15.5	41.7						
50–59	178	28.8	40.4						
60–69	180	29.1	38.9						
≥ 70	112	18.1	31.3						
Duration of dialysis (years) Mean ± *SD* 8.8 ± 5.7				1.032	1.020–1.044	< 0.0001*	1.032	1.017–1.042	< 0.0001*
Duration of dialysis (years)									
≥ 20	11	1.8	90.9						
10–19	83	13.4	83.1						
≤ 9	520	84.1	31.5						
Missing	4	0.7							
Hemodialysis at regional dialysis center									
Center 1	167	27.0	18.0	ref (1)		0.060	ref (1)		0.065
Center 2	63	10.2	69.8	1.440	1.138–1.822		1.436	1.135–1.817	
Center 3	78	12.6	33.3	1.130	0.891–1.433		1.134	0.894–1.439	
Center 4	153	24.8	50.3	1.274	1.054–1.541		1.271	1.051–1.537	
Center 5	45	7.3	35.6	1.149	0.862–1.531		1.163	0.871–1.552	
Center 6	48	7.8	35.4	1.148	0.867–1.519		1.151	0.870–1.524	
Center 7	64	10.4	53.1	1.298	1.019–1.654		1.302	1.022–1.659	
Hemodialysis at more than one center									
No	240	38.8	20.8	ref (1)		0.002*	ref (1)		0.026*
Yes	378	61.2	51.3	1.251	1.086–1.442		1.180	1.020–1.365	
Blood transfusion									
No	142	23.0	21.8	ref (1)		0.043*	ref (1)		0.341
Yes	476	77.0	44.7	1.188	1.006–1.404		0.92	0.817–1.041	

*SD* standard deviation, *PR* unadjusted prevalence ratio, *APR* adjusted prevalence ratio; *p < 0.05; *p* (Wald test)

Factors determined to be significantly associated with the anti-HCV positivity in the unadjusted Poisson analysis were as follows: age (prevalence ratio (PR) = 0.985, 95% confidence interval (CI): 0.972–0.998, *p* = 0.021), duration of hemodialysis (PR = 1.032, 95% CI: 1.020–1.044, *p* <  0.0001), hemodialysis at more than one center (PR = 1.251, 95% CI: 1.086–1.442, *p* = 0.002), and blood transfusion (PR = 1.188, 95% CI: 1.006–1.404, *p* = 0.043; Table 2). The multivariate analysis determined the duration of hemodialysis and hemodialysis at more than one center as independent significant factors associated with anti-HCV positivity (adjusted prevalence ratio (APR) = 1.032, 95% CI: 1.017–1.042, *p* <  0.0001; APR = 1.180, 95% CI: 1.020–1.365, *p* = 0.026; Table 2).

The duration of hemodialysis was significantly associated with HCV infection. Eleven patients with terminal chronic kidney insufficiency had started hemodialysis over 20 years earlier, of whom 10 (90.9%) were found to be anti-HCV positive in this study. In contrast, the majority of patients had started hemodialysis in the previous 9 years and had an anti-HCV positive rate of 31.5% (Table 2).

Patients that had received blood transfusion in the past had significantly higher anti-HCV prevalence (*p* = 0.043; Table 2). In addition, the number of blood transfusions received was significantly greater in the group of anti-HCV positive patients (*p* = 0.0013). The maximum number of blood transfusions received was 50 in the group of anti-HCV positive patients, and 20 in the group of anti-HCV negative patients (data not shown).

No statistically significant association of anti-HCV positivity with other possible risk factors (transmission among family members, surgical and dental interventions, tattoos, injecting drugs, piercing, using others’ shaving kits, imprisonment, and hemophilia) were found (data not shown).

The second part of the survey was directed toward the management of dialysis units and explored the reasons for nosocomial transmission of HCV. At the time the survey was carried out in 2015, a total of 18 medical doctors specialized in nephrology, and 129 nurses were employed at the hemodialysis centers in Kosovo, corresponding to one medical doctor tending 39 patients and one nurse tending five to six patients (Table 3).

**Table 3:** Resources and staff available at hemodialysis centers in Kosovo in December 2015

Hemodialysis center	Patients *n*	Patients per doctor *n*	Patients per nurse *n*	Dialysis machines *n*	Separate premises for HCV-positive patients	Sufficient supply of gloves	Sufficient supply of sterile gauze	Sufficient supply of disinfection material	Anti-HCV test available prior to start of dialysis
Center 1	185 (26.1%)	37	5.8	56	Yes	Yes	Yes	Yes	No
Center 2	70 (9. 9%)	70	5.8	18	Yes	Yes in last year, poor quality	Yes	Yes	No
Center 3	94 (13.3%)	47	6.3	22	No	Yes	No	Yes	No
Center 4	176 (24.9%)	44	6.5	37	No	No	No	No	No
Center 5	50 (7.1%)	50	5	30	Yes	Yes	Yes	Yes	Yes
Center 6	64 (9.0%)	16	4.3	20	Yes	Yes	Yes	No	Yes
Center 7	69 (9.7%)	69	3.8	22	Yes	Yes in last year, poor quality	No	No	No
Total	708	39	5.5	205					

Insufficient supply of gloves, sterile gauze, and disinfectants, as well as poor quality of gloves, were reported by several dialysis centers. Separate dialysis machines and staff were available for HCV-positive patients at all centers; however, separate premises were dedicated for isolation of HCV-positive patients at only five centers. Anti-HCV test results were available prior to admission for dialysis at two centers only (Table 3).

When questioned about possible reasons for high HCV prevalence in their hemodialysis centers, the management reported the following: human factors, surgical interventions, blood transfusion, lack of appropriate isolation premises, postwar shortage of supplies, inability to screen patients for HCV before dialysis is initiated, lack of HCV testing capacities, and inadequate disinfection of machines.

## Discussion

The results of this nationwide study showed a very high prevalence of HCV infection among patients undergoing hemodialysis at all centers in Kosovo in 2013. The overall HCV seroprevalence among hemodialysis patients in Kosovo was 53.0%, ranging from 22.3 to 91.1% at different centers. HCV seroprevalence exceeded 65.0% at four out of seven centers.

A comparison of these results with those from 2008 [13], showed that the overall seroprevalence of HCV infection in the hemodialysis setting in Kosovo significantly increased from 42.9 to 53.0% in 5 years (χ^2^, *p* = 0.0004). In contrast, a 2004 multicenter study showed that the prevalence of anti-HCV positive hemodialysis patients decreased markedly in one decade in the participating units from most European countries [17]. The latest data on HCV prevalence based on the cohort of patients surveyed in December 2015 again displayed a lower anti-HCV prevalence of 39.5%. The difference in the prevalence could be due to enforced prevention following the results of the 2013 study combined with the number of anti-HCV positive patients that died or migrated to western Europe in search of better treatment options. Notably, at the end of 2014 and the beginning of 2015 there was large-scale emigration of the Kosovo population, which could have affected this analysis [18].

The prevalence of HCV infection in hemodialysis patients in different countries in the region ranges from 12.7% in Serbia, 24.0% in Greece, 24.2% in Albania, and 32.0% in Macedonia to 58.9% in Bosnia and Herzegovina [19–23]. However, in contrast to our study, all the regional studies mentioned above assessed anti-HCV prevalence among hemodialysis patients originating from a single center, or a single region of a particular country, and so selection bias should be taken in account when interpreting and comparing data.

In contrast to hemodialysis patients, HCV seroprevalence in the general Kosovar population [11] and blood donors in Kosovo is relatively low [10], indicating that the high prevalence of HCV infection among hemodialysis patients is not a result of a high baseline prevalence of HCV infection in patients entering hemodialysis centers. Out of 73,295 blood donations reported between 2011 and 2013 in Kosovo, only 29 (0.04%) were identified as anti-HCV positive (data not published). Interestingly, the 2015 survey identified blood transfusion as a significant factor associated with HCV positivity in unadjusted statistical analysis. However, it is more likely that HCV was transmitted due to inappropriate infection control practices during the blood transfusion procedure at dialysis centers and not necessarily with contaminated blood products because all blood donors in Kosovo have been mandatorily screened for the presence of anti-HCV since 1992. Unfortunately, HCV RNA or HCV core antigen testing are still not part of the routine screening of donors in Kosovo, and so some HCV-positive individuals may have been missed. The use of blood transfusion among hemodialysis patients has declined significantly since the availability of erythropoietin in 2004 in Kosovo. In 2008, erythropoietin was available to all dialysis patients, and so in the later years blood transfusion could not have contributed to the transmission of HCV among dialysis patients to such an extent.

Nosocomial transmission of HCV among hemodialysis patients from Kosovo is further supported by the results of the 2015 survey, which identified the duration of hemodialysis and receiving dialysis at more than one center in Kosovo as independent factors significantly associated with anti-HCV positivity, and by the HCV genotype distribution determined in the 2013 study. Genotype 1a (62.2%) was the most frequent among hemodialysis patients in Kosovo, followed by genotypes 4d (33.1%), 1b (4.0%), and 2c (0.7%). HCV genotype 1a was predominant at all but one center, where genotype 4d prevailed (Table 1). Unfortunately, HCV genotype distribution in other groups of HCV-infected patients in Kosovo has not been studied so far, which prevents us from concluding that this genotype distribution pattern is specific to hemodialysis patients. However, according to data available in the literature, neither genotype 1a or 4d are predominant either in hemodialysis patients or in the HCV-infected population in general in any country in the region, except Kosovo [24]. In a study performed on 97 patients in neighboring Montenegro, both genotypes 1a and 4d were identified as the third most prevalent genotypes in HCV-infected patients with 19.6% share each [25], whereas in neighboring Macedonia HCV genotype 4 has not been identified among 1167 patients studied [26]. In contrast to Kosovo, where HCV genotype 1b was found in only 4.0% of hemodialysis patients and genotype 3 was not detected in a single hemodialysis patient, these two genotypes predominate among HCV-infected patients in general as well as hemodialysis patients in all other countries in the region [20, 25–31]. Further molecular epidemiology analysis of HCV strains from our hemodialysis patients, which is currently in progress, might elucidate the routes of HCV transmission at hemodialysis centers in Kosovo and irrevocably confirm our hypothesis that the high prevalence of HCV infection in hemodialysis patients in Kosovo is a result of nosocomial transmission.

The management of hemodialysis centers reported a number of issues that might have contributed to HCV transmission at dialysis centers. Most centers reported a shortage of staff, which directly relates to the quality of services provided at hemodialysis centers [32]. At almost all centers in Kosovo, the number of nephrologists and nurses was lower than needed in comparison to the number of patients undergoing dialysis [33]. Nephrologists from four centers did not work full-time at the hemodialysis units, but worked part-time at other internal disease units of the regional hospitals. In addition, none of the centers employed an infection control nurse and renal dialysis technician. In addition, centers reported limited space available to offer isolation for anti-HCV positive patients. However, even if isolation was possible, the problem remains that at most centers in Kosovo new hemodialysis patients did not have anti-HCV test results available prior to starting dialysis, and HCV RNA or HCV core antigen testing are still not performed in Kosovo today. The centers also struggled with the availability of gloves, disinfectants, and sterile gauze, and the overall absence of internal infection control guidelines.

A limitation of this study is that at the time of the initial HCV prevalence investigation in 2013 the demographic data were not available and interviews were conducted on a marginally different cohort of patients receiving hemodialysis in 2015, which had a significantly lower HCV prevalence. Another major limitation is that only anti-HCV positive and not anti-HCV negative patients were tested for the presence of HCV RNA. Because some studies reported a substantial proportion of HCV infected dialysis patients with undetectable anti-HCV antibodies in their serum, most likely due to immune dysfunction [34], the prevalence of HCV infection at hemodialysis centers in Kosovo might be underestimated.

## Conclusions

In conclusion, a nationwide study of all hemodialysis centers in Kosovo showed an extremely high prevalence of HCV infection in hemodialysis patients. The relatively low prevalence of HCV infection in the general Kosovar population, the significant increase in the anti-HCV prevalence among hemodialysis patients in recent years, the predominance of two otherwise rare HCV genotypes in the region, HCV infection associated with a longer duration of hemodialysis, and hemodialysis at more than one center indicate nosocomial transmission due to inappropriate infection control practices as the main HCV transmission route. In most European countries, consistent reinforcement of hygienic precautions and/or isolation strategies in hemodialysis units has resulted in a substantial decrease of both the incidence and prevalence of HCV infection in hemodialysis units [35]. It seems that the WHO goal of eliminating HCV by 2030 might be difficult to achieve in settings similar to Kosovo if more rigorous measures are not implemented for effective control of HCV infection in the hemodialysis environment [36].

## Additional files

Additional file 1: **Table S1**. Questionnaire used in the face-to-face interviews with patients on hemodialysis in 2015. (DOCX 23 kb)

Additional file 2: **Table S2**. Questionnaire used in the face-to-face interviews with the management of hemodialysis centers. (DOCX 14 kb)

## Abbreviations

APR: Adjusted prevalence ratio
CI: Confidence interval
HCV: Hepatitis C virus
PCR: Polymerase chain reaction
PR: Prevalence ratio
RNA: Ribonucleic acid
RT-PCR: Reverse transcription polymerase chain reaction
*SD*: Standard deviation
WHO: World Health Organization
