# Supplementary material for: A very high prevalence of hepatitis C virus infection among patients undergoing hemodialysis in Kosovo: a nationwide study
Source: BMC Nephrol. 2018 Nov 3;19:304. doi: 10.1186/s12882-018-1100-5 (PMC6215601; doi:10.1186/s12882-018-1100-5)
Supplement: Supplementary file 1 — Table S1. Questionnaire used in the face-to-face interviews with patients on hemodialysis in 2015. (DOCX 23 kb) [file 12882_2018_1100_MOESM1_ESM.docx]

**Table S1. Questionnaire used in the face-to-face interviews with patients on hemodialysis in 2015**

|  |  |
| --- | --- |
|  | Interviewer: |
|  | Time of questionnaire initiation: |
|  | |
| **1.** | **First and last name of the dialysis patient:** |
|  |  |
|  | |
| **2.** | **Date of birth:** |
|  |  |
|  | |
| **3.** | **Sex:** |
| a) | Female |
| b) | Male |
|  | |
| **4.** | **Address:** |
| a) | Residency: |
| b) | Municipality: |
|  | |
| **5.** | **Marital status:** |
| a) | Single |
| b) | In relationship |
| c) | Engaged |
| d) | Married |
| e) | Widowed |
| f) | Divorced |
|  | |
| **6.** | **Education:** |
| a) | No formal education |
| b) | Elementary school |
| c) | High school |
| d) | University |
| e) | Other:__________________ |
|  | |
| **7.** | **Profession:** |
| a) |  |
| b) |  |
| c) |  |
|  | |
| **8.** | **Employment status:** |
| a) | Unemployed |
| b) | Employed in a public sector |
| c) | Employed in a private sector |
| d) | Retired |
|  | |
| **9.** | **Have you ever heard about hepatitis C virus (HCV)?** |
| a) | Yes |
| b) | No (IF NO, GO TO QUESTION 11) |
| c) | Don't know |
|  |  |
| **10.** | **Do you know how HCV is transmitted?** |
| a) | Yes |
| b) | No |
| c) | Don't know |
|  |  |
| **11.** | **Have you ever been diagnosed with HCV infection?** |
| a) | Yes |
| b) | No (IF NO, GO TO QUESTION 13) |
| c) | Don't know |
|  | |
| **12.** | **When have you been diagnosed with HCV infection (year)?** |
|  |  |
|  |  |
| **13.** | **Have you ever had skin jaundice?** |
| a) | Yes |
| b) | No |
| c) | Don't know |
|  |  |
| **14.** | **Have any of your family members ever had HCV infection?** |
| a) | Yes |
| b) | No (IF NO, GO TO QUESTION 16) |
| c) | Don't know |
|  | |
| **15.** | **Who of your family members has (or had) HCV infection?** |
| a) | Father |
| b) | Mother |
| c) | Wife/Husband |
| d) | Daughter |
| e) | Son |
| f) | Other: |
|  | |
| **16.** | **Have you ever received blood transfusion?** |
| a) | Yes |
| b) | No (IF NO, GO TO QUESTION 20) |
| c) | Don't know |
|  | |
| **17.** | **In what year did you receive the first blood transfusion?** |
|  |  |
|  | |
| **18.** | **How many blood transfusions have you received up to now?** |
|  |  |
|  | |
| **19.** | **Where did you receive blood transfusions? Please list health institution(s).** |
| a) |  |
| b) |  |
| c) |  |
|  | |
| **20.** | **Have you ever used dental services?** |
| a) | Yes |
| b) | No (IF NO, GO TO QUESTION 22) |
| c) | Don't know |
|  | |
| **21.** | **List the dental services and the names of medical institutions where these services were provided.** |
|  | Type of dental service: |
| a) |  |
| b) |  |
| c) |  |
|  | Name of institution: |
| a) |  |
| b) |  |
| c) |  |
|  | |
| **22.** | **Have you ever had surgical intervention?** |
| a) | Yes |
| b) | No (IF NO, GO TO QUESTION 24) |
| c) | Don't know |
|  | |
| **23.** | **List the surgical interventions and the names of medical institutions where these interventions took place.** |
|  | Type of surgical intervention: |
| a) |  |
| b) |  |
| c) |  |
|  | Name of health institution: |
| a) |  |
| b) |  |
| c) |  |
|  | |
| **24.** | **Have you ever undergone dialysis?** |
| a) | Yes |
| b) | No (IF NO, GO TO QUESTION 29) |
| c) | Don't know |
|  | |
| **25.** | **In what year did you start with dialysis?** |
|  |  |
|  | |
| **26.** | **At which center did you receive dialysis for the first time? *** |
| a) | Center 1 |
| b) | Center 2 |
| c) | Center 3 |
| d) | Center 4 |
| e) | Center 5 |
| f) | Center 6 |
| g) | Center 7 |
| h) | Other: |
|  | |
| **27.** | **At which centers have you received dialysis services? Multiple answers possible. *** |
| a) | Center 1 |
| b) | Center 2 |
| c) | Center 3 |
| d) | Center 4 |
| e) | Center 5 |
| f) | Center 6 |
| g) | Center 7 |
| h) | Other: |
|  | |
| **28.** | **How often are you undergoing dialysis?** |
| a) | Once a week |
| b) | Twice a week |
| c) | Three times a week |
| d) | Other: |
|  | |
| **29.** | **Have you ever done any of the following:** |
| a) | Tattoo |
| b) | Ear or other body piercing |
| c) | Injected drugs |
| d) | Needle stick injury at the work place |
| e) | Shared shaving kit with somebody |
| f) | Shared toothbrush with somebody |
| g) | Shared the toilet with an HCV-positive family member |
| h) | Organ transplantation |
| i) | Extra-marital sexual relations |
|  | |
| **30.** | **Have you ever been imprisoned?** |
| a) | Yes |
| b) | No (IF NO, GO TO QUESTION 33) |
| c) | Don't know |
|  | |
| **31.** | **Where were you imprisoned? List the centers.** |
| a) |  |
| b) |  |
| c) |  |
|  |  |
| **32.** | **How long was the prison sentence?** |
|  |  |
|  | |
| **33.** | **Have you been diagnosed with haemophilia?** |
| a) | Yes |
| b) | No |
| c) | Don't know |
|  | |
| **34.** | **Have you ever been treated with interferon/ribavirin?** |
| a) | Yes |
| b) | No (IF NO, GO TO THE QUESTION 40) |
|  | |
| **35.** | **When did you initiate interferon/ribavirin treatment?** |
|  |  |
|  | |
| **36.** | **Where did you initiate interferon/ribavirin treatment?** |
|  |  |
|  | |
| **37.** | **Who was the medical doctor responsible for your interferon/ribavirin treatment?** |
|  |  |
|  | |
| **38.** | **Did you complete the interferon/ribavirin treatment?** |
| a) | Yes |
| b) | No |
| c) | Don't know |
|  | |
| **39.** | **What where the main complaints related with the intake of interferon/ribavirin?** |
| a) |  |
| b) |  |
| c) |  |
| d) |  |
| e) |  |
| f) | I didn’t have any complaints during the treatment. |
|  | |
| **40.** | **What were the reasons for not enrolling in the interferon/ribavirin treatment?** |
| a) | Didn’t want to undergo treatment |
| b) | No available spots in the treatment program |
| c) | Couldn’t pay for the treatment myself |
| d) | Didn’t know that treatment for HCV infection exists |
| e) | Don’t know |
|  | |
|  | **Possible final remarks by the patient:** |
|  |  |
|  |  |
|  |  |
| **THANK YOU FOR YOUR TIME AND COOPERATION** | |
|  |  |
|  | Time of interview completion: |

*Names of the dialysis centers were anonymized for the purpose of this study.
