# Supplementary material for: A very high prevalence of hepatitis C virus infection among patients undergoing hemodialysis in Kosovo: a nationwide study
Source: BMC Nephrol. 2018 Nov 3;19:304. doi: 10.1186/s12882-018-1100-5 (PMC6215601; doi:10.1186/s12882-018-1100-5)
Supplement: Supplementary file 2 — Table S2. Questionnaire used in the face-to-face interviews with the management of hemodialysis centers. (DOCX 14 kb) [file 12882_2018_1100_MOESM2_ESM.docx]

**Table S2. Questionnaire used in the face-to-face interviews with the management of hemodialysis centers**

|  | |
| --- | --- |
| **1** | **How many medical doctors are currently employed at the dialysis center?** |
|  |  |
|  | |
| **2** | **How many nurses are currently employed at the dialysis center?** |
|  |  |
|  | |
| **3** | **How many dialysis machines are utilized at the center?** |
|  |  |
|  | |
| **4** | **How many square meters does the dialysis center have?** |
|  |  |
|  | |
| **5** | **How many patients in total are receiving dialysis services at the center?** |
|  |  |
|  | |
| **6** | **Do you have separate dialysis machines for HCV positive patients?** |
| a) | Yes |
| b) | No |
| c) | Don't know |
|  | |
| **7** | **Do you have separate premises for HCV positive patients?** |
| a) | Yes |
| b) | No |
| c) | Don't know |
|  | |
| **8** | **Do you have specific staff dedicated to HCV positive patients only?** |
| a) | Yes |
| b) | No |
| c) | Don't know |
|  | |
| **9** | **Are dialysis machines maintained regularly?** |
| a) | Yes |
| b) | No |
| c) | Don't know |
|  | |
| **10** | **Do you always have sufficient supply of gloves for your daily work?** |
| a) | Yes |
| b) | No |
| c) | Don't know |
|  | |
| **11** | **Do you always have sufficient supply of sterile gauzes for your daily work?** |
| a) | Yes |
| b) | No |
| c) | Don't know |
|  | |
| **12** | **Do you always have sufficient supply of disinfection material for your daily work?** |
| a) | Yes |
| b) | No |
| c) | Don't know |
|  | |
| **13** | **Do you always have patients’ anti-HCV results available before dialysis initiation?** |
| a) | Yes |
| b) | No |
| c) | Don't know |
|  | |
| **14** | **At which institution is HCV testing for your dialysis patients usually performed?** |
| a) | National Institute of Public Health of Kosovo |
| b) | Blood transfusion |
| c) | Private laboratories |
| d) | Other: |
|  | |
| **15** | **In your opinion, what are the main reasons for the high prevalence of HCV infection in your dialysis unit?** |
|  |  |
|  |  |
| **THANK YOU FOR YOUR TIME AND COOPERATION** | |
|  |  |
| Date and time of interview completion: | |
